# Adolescent-like Processing of Behaviorally Salient Cues in Sensory and Prefrontal Cortices of Adult Preterm-Born Mice

**DOI:** 10.21203/rs.3.rs-5529783/v1

**Published:** 2024-12-11

**Authors:** Adema Ribic, Emily McCoy, Vyshnavi Pendala, Mona Fariborzi, Lara Demir, Olivia Buell, Samuel Fedde, Jacqueline Stinger, Luciano Elbaum, Troy Holsworth, Philip Amenyo Awude

**Affiliations:** University of Virginia; University of Virginia; University of Virginia; University of Virginia; University of Virginia; University of Virginia; University of Virginia; University of Virginia; University of Virginia; University of Virginia; University of Virginia

**Keywords:** preterm, prefrontal, visual, task, representation, processing

## Abstract

Preterm birth is a leading risk factor for atypicalities in cognitive and sensory processing, but it is unclear how prematurity impacts circuits that support these functions. To address this, we trained adult mice born a day early (preterm mice) on a visual discrimination task and found that they commit more errors and fail to achieve high levels of performance. Using *in vivo electrophysiology*, we found that the neurons in the primary visual cortex (V1) and the V1-projecting prefrontal anterior cingulate cortex (ACC) are hyper-responsive to the reward, reminiscent of cue processing in adolescence. Moreover, the non-rewarded cue fails to robustly activate the V1 and V1-projecting ACC neurons during error trials, in contrast to prefrontal fast-spiking (FS) interneurons which show elevated error-related activity, suggesting that preterm birth impairs the function of prefrontal circuits for error monitoring. Finally, environmental enrichment, a well-established paradigm that promotes sensory maturation, failed to improve the performance of preterm mice, suggesting limited capacity of early interventions for reducing the risk of cognitive deficits after preterm birth. Altogether, our study for the first time identifies potential circuit mechanisms of cognitive atypicalities in the preterm population and highlights the vulnerability of prefrontal circuits to advanced onset of extrauterine experience.

## Introduction

Children born prematurely (< 37 weeks of gestation) are at 4–5x higher risk for developing intellectual disability (ID) and attention-deficit/hyperactivity disorder (ADHD) ^1–3^, at 10x higher risk for developing autism spectrum disorder (ASD) ^4,5^, and display persistent deficits in executive function and academic performance ^6–8^. As preterm labor occurs in > 10% of all births in US and worldwide, preterm birth is the leading risk factor for cognitive and neurodevelopmental conditions ^6,9–11^. Yet, the neural circuits whose function may be disrupted by preterm birth are still unknown.

Impairments in the structure and function of the frontal cortex are frequently associated with executive dysfunction and neurodevelopmental conditions with high incidence in the preterm population ^9,12^. Unsurprisingly, neuroimaging and neurophysiological studies in preterm infants and children revealed reduced volume of and connectivity between frontal, thalamic and sensory areas ^11,13–16^. On a cellular level, studies of postmortem prefrontal cortex samples obtained from preterm fetuses demonstrated reduced density of inhibitory interneurons and altered expression of genes related to inhibitory neurotransmission ^17,18^. Some of the changes in brain structure can be recapitulated in animal models of prematurity, which also display disrupted layer-specific distribution and proportion of cortical interneurons ^18–21^, and alterations in the expression of multiple neurotransmission markers ^22–24^. Behaviorally, animal models of prematurity often display persistent hyperactivity ^23,25–27^, and some cognitive deficits ^18,28^, but the link between preterm birth-related changes in the frontal cortex and high incidence of cognitive dysfunction in the preterm population remains unclear.

To address this, we performed a detailed neurophysiological study of prefrontal function in adult mice born prematurely that were trained on a prefrontal cortex (PFC)-dependent visual discrimination task. While adult mice born 1 day early do not display any changes in baseline visual processing and locomotor activity, they show significantly reduced discriminability and response inhibition, indicative of executive dysfunction. Electrophysiologically, prefrontal and visual cortices of preterm mice have heightened responses to the visual cue associated with reward, reminiscent of reward representation in adolescent brain ^29–31^, and visual cortex-projecting prefrontal neurons show weakened representation of the non-rewarded cue during error trials. In contrast, optogenetically and electrophysiologically identified prefrontal fast-spiking interneurons have blunted responses to the rewarded cue and elevated activity during the error trials, providing for the first time functional evidence of inhibitory dysfunction in the preterm PFC. Surprisingly, postnatal environmental enrichment-a classical paradigm for improving the outcomes of impaired neurodevelopment ^21,32–36^-failed to improve the task performance in preterm mice, suggesting limited efficacy of early interventions in improving negative neurodevelopmental outcomes after preterm birth ^37^. Together, our study demonstrates circuit- and cell-type specific impairments in prefrontal function and top-down sensory processing after preterm birth, uncovering the potential circuit mechanism of cognitive dysfunction in preterm born population.

## Methods

### Mice.

Mice were maintained on C57BL/6 background (The Jackson Laboratory, Bar Harbor, ME) on reverse 12:12 light:dark cycle (lights off 11 AM-11 PM), with food and water *ad libitum*, except during visual discrimination training (see below). Animals of both sexes were used between 2 and 7 months of age. Preterm mice were generated through timed breedings, where the day after the pairing was considered as gestational day (GD) 0. Once the pregnancy was confirmed (> 1.5g increase in weight at GD 10), pregnant dams were habituated to handlers by daily handling sessions. Mifepristone (MFP, Milipore Sigma, Burlington, MA or HelloBio, Bristol, UK) was dissolved in DMSO (Milipore Sigma) and 150 μg was injected subcutaneously on GD 17. Preterm mice were delivered on GD 18. The cage with preterm mice was occasionally supplemented with external heat and oxygen to prevent hypothermia and hypoxia, commonly observed in preterm mice. Control term mice were obtained from timed pregnant dams injected with DMSO on GD 17, and with MFP on GD 18. All dams received continuous enrichment in form of plastic igloos and nesting material, as well as sunflower seeds (Bio-Serv, Flemington, NJ) beginning 3–4 days before parturition. Animals were treated in accordance with the University of Virginia Institutional Animal Care and Use Committee guidelines.

### Environmental enrichment.

Nursing dams with their litters were transferred to a standard rat cage when the pups reached postnatal day (P) 5. Dams were co-housed with a nursing dam and its litter that was age matched and on BALB/c strain (purchased from Jackson Laboratory) to facilitate social enrichment. Cages contained multiple enrichment objects: InnoDome and InnoWheels from BioServ, gnawing sticks, multiple nesting pads, shredded paper and hay for building nests, as well as a variety of chew toys (pumice chews, willow twigs, willow balls, veggie chews, and hay tunnels, hideaways and sticks). Large enrichment objects were rearranged in the cage every 2 days, while chew toys were replaced with new ones ones every 2–3 days. Once the litters reached P25, they were weaned and group-housed in standard cages at 2–3 mice/cage to allow for placement of an InnoDome and InnoWheel, as well as 2–3 small chew toys. When the mice reached P60, they were prepared for visual discrimination task as described below.

### Viruses.

Following viruses were purchased from Addgene: AAV-S5E2-ChR2-mCherry (135634-AAV1; 2×10^13^ vg/ml), AAV-EF1a-double floxed-hChR2(H134R)-mCherry-WPRE-HGHpA (20297-AAV9; 3.2×10^13^ vg/ml) and AAV-hSyn-HI-eGFP-Cre-WPRE-SV40 (105540-AAVrg; 1.7×10^13^ vg/ml). All viruses were diluted 1:10 in sterile PBS before the injection.

### Surgeries.

Animals were anesthetized with isoflurane in oxygen (2–2.5% induction, 1–1.5% maintenance), warmed with a heating pad at 38°C and given subcutaneous injections of Buprenorphine SR (1 mg/kg) or Rimadyl (5 mg/kg) and 0.25% Bupivacaine (beneath their scalp). Eyes were covered with Puralube (Decra, Northwich, UK). Scalp and fascia from Bregma to behind lambda were removed, and the skull was cleaned, dried and covered with a thin layer of Scotchbond adhesive (3M, Maplewood, MN). Skin edges were sealed with VetBond (3M).

For virus injections, the head was immobilized in a custom-made stereotactic apparatus and small craniotomies were made on the left hemisphere with a dental drill at the following coordinates: primary visual cortex (V1): 0.5 mm anterior to lambda, 2–3 mm lateral to midline; anterior cingulate cortex (ACC): 2–2.5 mm anterior to bregma, 0.2–0.5 mm lateral to midline. NanoFil syringe (35 G beveled needle; WPI, Sarasota, FL) was then used to inject the virus at 1 nl/s using a Syringe Pump (KD Scientific, Holliston, MA). Volume of injected virus was 300 nl, injected in 2 150 nl increments at 2 different depths: 500 and 250 μm beneath the brain surface for V1 (targeting layers 2/3 and 5), and 700 and 800 μm for the PFC. The syringe was left in place for 5–10 minutes after the injection to allow the virus solution to diffuse and then very slowly raised to the surface to prevent the backflow. Muscimol (TMR-X conjugate; HelloBio, Princeton, NJ) was injected in the same manner, volume and at the same coordinates of trained term mice at 1.6 mM concentration ^38^.

Head plates were attached after the Scotchbond adhesive application for animals that received no virus injection, or immediately after the virus injection for those that did. The head plate (stainless steel, SendCutSend, Reno, NV) was attached with dental cement (RelyX Ultimate, 3M). After the cement cured, the well of the head plate was filled with silicone elastomer (Reynold Advanced Materials, Brighton, MA) to protect the skull.

If the mice received Rimadyl for analgesia, they were given Rimadyl dissolved in hydrogel (1% food-grade agar in distilled water) during the recovery ^39^. Animals were group-housed after the implantation and monitored daily for signs of shock or infection. On the day of electrophysiological recording, the animals were anesthetized as above and craniotomies (~ 0.5 mm in diameter) were made above V1, PFC and cerebellum with 18G needles. If mice were injected with viruses, the existing craniotomies were reopened. The brain surface was covered in 2–3% low melting point agarose (Promega, Madison, WI) in sterile saline and then capped with silicone elastomer. Animals were allowed to recover for 1.5–2 h before the recording.

### Electrophysiology and optogenetics.

For the recording sessions, mice were placed in the head-plate holder above an aluminum mesh treadmill and allowed to habituate for 5–10 minutes. The silicone plug was removed, the reference (insulated silver wire electrode; A-M Systems, Carlsborg, WA) was placed in cerebellum and the well was covered with warm sterile saline. A multisite electrode spanning all cortical layers (A1×16–5mm-50–177-A16; Neuronexus Technologies, Ann Arbor, MI) was coated with DiI (Invitrogen) to allow post hoc insertion site verification and then inserted in the V1 through the craniotomy. For PFC, a 4×4 shank probe (A4×4–3mm-50–125-177-A16) was used for the recordings, coated with DiI, DiO or DiD based on the type of fluorophore used in the experiment (mCherry or EGFP; listed in Viruses). The electrodes were slowly (5–10 μm/s) lowered to the appropriate depth: 800 μm for the PFC and until the uppermost recording site had entered the brain for the V1 and allowed to settle for 15–30 minutes. For optogenetics, a 473 nm laser diode with 0.4 μm fiber tip (0.22 NA, Doric Lenses, Quebec, Canada) was lowered to the craniotomy. 10 ms square pulses were delivered at 3–5 mW/mm^2^ (200 pulses at 0.5 Hz) using Spike2 (CED, Cambridge, UK) after the last recording session for optogenetic identification of neuronal subtypes (optotagging). The well was filled with 3% agarose prior to the recordings to stabilize the electrode and the whole region was kept moist with surgical gelfoam soaked in sterile saline (Pfizer, MA). The signals from the recording probes were fed into a 16-channel amplifier (Model 3500; A-M Systems, Sequim, WA) and amplified 200x, before being sampled at 25 kHz using Spike2 and Power 1401–3 data acquisition unit (CED). After the recording ended, the electrodes were slowly retracted, and the well was cleaned and protected with silicone elastomer.

### Visual stimuli.

For visual stimuli, blank screen was generated with MATLAB (MathWorks, Natick, MA) using the PsychToolBox extension (Brainard, 1997) and presented on a gamma corrected 27” LCD. The screen was centered 20–25 cm from the mouse’s right eye, covering ∼80° of visual space. For calculating baseline orientation selectivity, 12 orientations 30º apart were presented at 100% contrast at 0.15 cpd, with 1 s long stimuli and 1 s interstimulus interval (blank screen). For visual discrimination training, visual stimuli (120º and 240º) were presented at 0.15 cpd and 100% contrast in a random sequence for 3 seconds, followed by a 15 second long interstimulus interval (blank screen). Presentation of 120º triggered the delivery of 10 μl of water through a syringe pump (New Era Pump Systems, Farmingdale, NY), while the presentation of 240º had no consequence.

### Behavior.

4–7 days after the headpost implantation, mice were gradually water restricted (from 3 ml of water/day to 1 ml of water/day) for 1 week. Food was available *ad libitum*. Mice were weighed daily and their weight was maintained at 85% of initial weight to prevent dehydration. During water restriction, mice were gradually habituated to handling by the experimenters, the treadmill, and the water delivery spout during daily habituation sessions ^40^. After 1 week of water restriction, the visual discrimination training began, where mice were headfixed above the treadmill and a stainless-steel gavage needle (18G) was positioned near the mouse’s mouth for water delivery. The pump was controlled by the Power1401–3 data acquisition interface (CED) for lick detection ^41^. Mice were trained in 2 daily sessions consisting of 50 120º and 240º presentations each, for a total of 200 trials per day. Licks of the spout during the stimulus presentation, as well as during the first 10 s of the interstimulus interval were quantified according to the signal detection theory as Hits (the fraction of trials in which the mouse licked to the 120º orientation), Misses (the fraction of trials in which the mouse failed to lick to the 120º orientation), False Alarms (the fraction of trials in which the mouse licked to the 240º orientation) and Correct Rejections (the fraction of trials in which the mouse failed to lick to the 240º orientation), where the performance was measured as discriminability (d’) = z(H) - z(FA). Mice were trained until they achieved a d’ of 2 or above for 3 consecutive sessions.

### Electrophysiological and behavioral data analysis.

Spikes were isolated from the recordings using template matching in Spike2 (CED). Briefly, the recordings were band-pass filtered (0.7–7 kHz) and smoothed (1 ms). Threshold for detection was set at 3 SDs of the mean baseline (blank screen) and a window of 0.9–1 ms. Isolated units were clustered using waveform properties (amplitude, spike half-width and slope of repolarization), and the clusters were checked manually for quality in addition to confirming there were no spikes during the refractory period (2 ms) using interval histograms. For orientation selectivity, custom-written scripts in Spike2 were used to plot the baseline-subtracted response of isolated units to the 12 orientations (10 ms bin) and construct a tuning curve, which was then fitted with a double Gaussian to determine the preferred (O_pref_) and orthogonal (O_orth_)orientations, and calculate the orientation selectivity index (OSI) as (R_pref_-R_orth_)/(R_pref_+R_orth_), where R is the firing rate at preferred and orthogonal orientations. Tuning width was calculated as half-width at half-maximum of the O_pref_.

For optotagging, spikes were isolated from optogenetic recording sessions and PSTHs (0.1–1 ms bin) were constructed in response to the laser pulses. Units with reliable responses (> 50%) of trials and short latencies (within 10 ms of the laser pulse) were considered optotagged and their waveforms were used to identify their spiking during the task performance. Unit waveforms were further compared between optotagging and task recordings for quality assurance.

Modulation indices (MIs) were calculated from 10 ms peristimulus time histogram (PSTHs) as (R_cue_- R_baseline_)/(R_cue_+R_baseline_), where R_var_ is the firing rate during the initial 1–1.5s presentation of 120º and 240º inside and outside of the task, as well as during different behavioral outcomes isolated using custom scripts in Spike2. Significantly modulated cells were calculated by comparing the R_cue_ and R_baseline_ using a t-test. If baseline firing rates between the groups were not statistically different, PSTHs were converted to z-scored values before comparing area under the curve (AUC) values.

Behavioral responses during the task were analyzed using custom scripts in Spike2 by converting the detected licks into events and constructing PSTHs in response to both stimuli.

### Histology and imaging.

Mice were anesthetized with a mixture of ketamine and xylazine and transcranially perfused with warm 0.1 M phosphate buffer, followed by warm 4% paraformaldehyde (Electron Microscopy Sciences, Hatfield, PA). Brains were postfixed 1 hr at room temperature, followed by overnight fixation at 4°C. Brains were sectioned into 40–80 μm sections using a vibratome and stored in 1x phosphate buffered saline (PBS) and 0.01% sodium azide. For immunohistochemistry, sections were rinsed in PBS, non-specific binding was blocked with 3% normal horse serum (heat inactivated, ThermoFisher, Waltham, MA) and 0.3% Triton-X 100 (Sigma-Aldrich) in PBS (sterile filtered). Antibodies were incubated overnight at 4°C. Parvalbumin (anti-goat) was used at 1/200 (Swant, Belinzona, Switzerland) and detected with donkey anti-goat Alexa 647 (ThermoFisher). After staining, sections were rinsed in distilled water, mounted on glass slides, briefly dried, and coverslipped with Aquamount (Polysciences, Warrington, PA). Images were acquired using Leica Stellaris 8 at 2048×2048 resolution using 10x HC PLAN FLUOTAR air (NA = 0.3 for tiling) or 40x HC PL APO CS2 (NA = 1.3) oil immersion objective.

### Quantification and statistical analysis.

All analyses were performed with the researchers blind to the condition. Statistical analyses were performed in Spike 2, Microsoft Excel or GraphPad Prism 9.0 (GraphPad Inc., La Jolla, USA) using as indicated in text and figure legends. All data are reported as mean ± SEM, where N represents the number of animals or the number of single units, as indicated. Target power for all sample sizes was 0.8 and alpha was set to 0.05.

## Results

### Adult preterm mice commit more errors during visual discrimination

**Figure 1:**
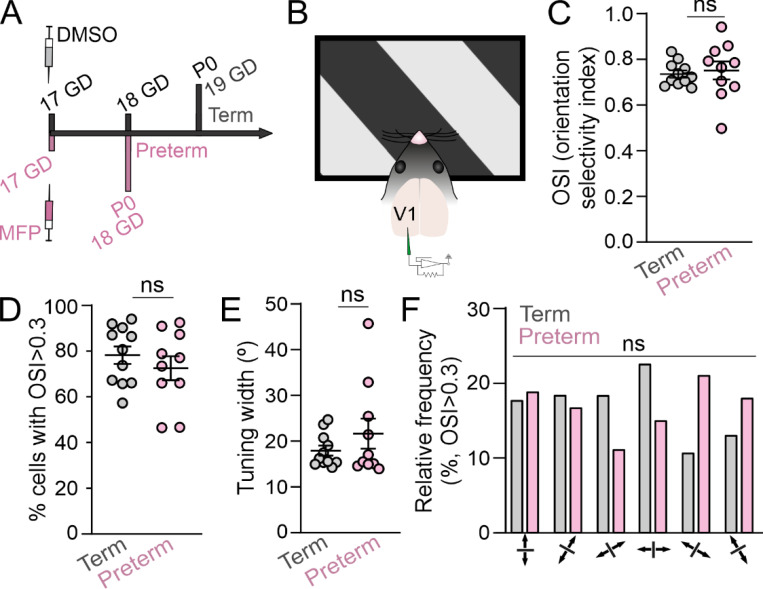
Adult preterm mice have intact orientation selectivity. **A)** Preterm mice were generated through subcutaneous injection of mifepristone (MFP) to timed pregnant dams at gestational day (GD) 17. Preterm mice were delivered at GD18, a day early. Control term mice were delivered at GD19 by dams injected with vehicle (DMSO) at GD17. **B)** Primary visual cortex (V1) of mice was recorded while mice were presented with 12 different orientations 30º apart. Preterm mice had no significant differences in **C)** orientation selectivity index (OSI: Term=0.74±0.02, Preterm=0.74±0.04, Welch’s t-test p=0.83, t=0.23, df=11.82;), **D)** fraction of neurons with OSI<0.3 (% selective neurons: Term=77.59±3.81, Preterm=72.04±5.22, t-test p=0.4, t=0.87, df=19), **E)** tuning width of selective neurons (Term=17.96±1.1, Preterm=21.57±3.3, Welch’s t-test p=0.32, t=1.04, df=10.99), or **F)** the distribution of preferred frequencies (p=0.19, X^2^ test). N=414 neurons from 11 term mice and 306 neurons from 10 preterm mice.

Children born preterm have an increased rate of cognitive deficits and neurodevelopmental conditions with unclear circuit origins ^9^. As we previously found that preterm birth similarly impacts the resting neural activity in the primary visual cortex (V1) of juvenile mice and infants ^24^, we hypothesized that mice born preterm would also display cognitive deficits. To test this, we chose to train the mice in a visual discrimination task, a classical learning task that facilitates electrophysiological probing of neural circuits in awake, behaving mice ^42,43^. Preterm mice were generated through subcutaneous injections of mifepristone (MFP) to timed-pregnant C57BL/6 dams on gestational day (GD) 17 (Fig. 1A), resulting in birth of viable preterm pups on GD 18. Term pups in our colony are born on GD 19 with very little variability, as previously published ^44,45^. Before training, adult (2–7 months old) male and female term and preterm mice were implanted with headposts for head fixation and their baseline visual function in form of orientation selectivity was tested using *in vivo* electrophysiology ^46^. We collected neuronal responses to sinusoidal oriented gratings (6 orientations 30º apart), at 100% contrast and 0.15 cycles per degree spatial frequency; Fig. 1B), and determined the orientation selectivity index (OSI; Fig. 1C), the number of orientation selective neurons (Fig. 1D), orientation tuning width (Fig. 1E), and the distribution of preferred frequencies ^47^. While neurons in the V1 of preterm mice had mildly elevated baseline firing rates (during the presentation of the blank screen; Term = 6.86 ± 0.53 spikes/s; Preterm = 10.32 ± 0.7 spikes/s, p = 0.064, nested t-test, 414 neurons from 11 term mice and 306 neurons from 10 preterm mice), we found no significant differences in any of the orientation selectivity measures between term and preterm mice (OSI: Term = 0.74 ± 0.02, Preterm = 0.74 ± 0.04, Welch’s t-test p = 0.83, t = 0.23, df = 11.82; % selective neurons: Term = 77.59 ± 3.81, Preterm = 72.04 ± 5.22, t-test p = 0.4, t = 0.87, df = 19; tuning width (º): Term = 17.96 ± 1.1, Preterm = 21.57 ± 3.3, Welch’s t-test p = 0.32, t = 1.04, df = 10.99; N = 11 term and 10 preterm mice; distribution of orientation preferences p = 0.19, X^2^ test). Our results indicated that preterm birth in mice does not negatively impact baseline orientation selectivity and that they can be trained in an orientation discrimination task.

**Figure 2:**
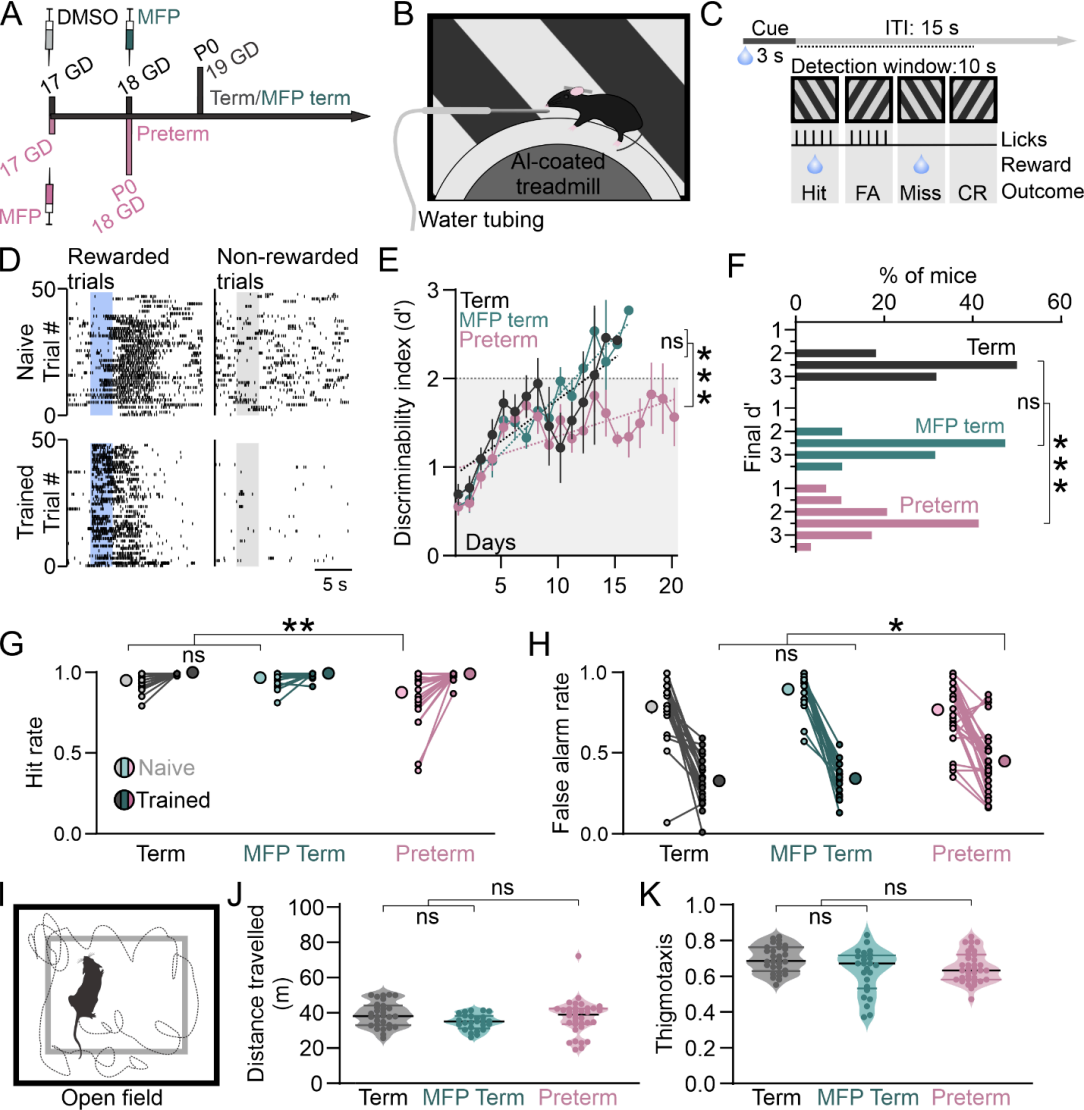
Preterm mice show impaired discriminability and commit more errors during visual discrimination. **A)** Schematics of gestation length for experimental groups. Preterm mice were generated through subcutaneous injection of MFP to timed pregnant dams at GD17, and control term mice were delivered at GD19 by dams injected with vehicle (DMSO) at GD17 or with MFP at GD18. **B)** Head-fixed term and preterm mice were trained to discriminate two orientations while locomoting on a treadmill positioned in front of a screen displaying the task cues. The reward (water) was delivered through a spout positioned near the mouth. **C)** Task structure: a single session consisted of 50 randomized, 3 s long presentations of rewarded and non-rewarded cue each, with an intertrial interval of 15 s. The detection window was 10 s. Four possible outcomes are Hit, False Alarm (FA), Miss and Correct Rejection (CR), as indicated. **D)** Representative lick raster of naïve (top) and trained (bottom) mice to the rewarded (left) and non-rewarded (right) cues, indicated with a blue and grey rectangle, respectively. Note the reduced reaction times after the presentation of the rewarded cue and withholding of licks during and after the non-rewarded cue in trained mice. **E)** Behavioral performance was measured as d’, which showed linear improvements in both groups of term mice (linear regression: R^2^ Term=0.73, MFP Term=0.93; p=0.32, F=0.97, DFn=1, DFd=354). Learning trajectory of preterm mice, however, had a significantly fatter slope, indicating impaired learning (R^2^ Preterm=0.48; p<0.0001, F=16.92, DFn=2, DFd=43; N=22 Term/19 MFP Term and 29 Preterm mice). **F)** A significant fraction of preterm mice (17.24%) failed to reach the criterion, in contrast to both groups of term mice (p<0.0001, **Χ**^2^ test). **G)** Hit rates of naïve preterm mice are significantly lower at the onset of training (2-way ANOVA birth x training p=0.004, F_(1, 68)_=9.16; birth p=0.003, F=9.25; training p<0.0001, F=36.88; Sidak post hoc: Term/MFP Term vs Preterm naïve p<0.0001, trained p=0.9). **H)** False Alarm rates were significantly higher in trained preterm mice, indicating impaired response inhibition (2-way ANOVA birth x training p=0.002, F_(1, 68)_=10.30; birth p=0.44, F=0.6; training p<0.0001, F=203.8; Sidak post hoc: Term/MFP Term vs Preterm naïve p=0.19, trained p=0.01). **I)** Schematics of the open field test. Mice were released into an open arena and their locomotor activity was recorded and quantified. **J)** Term and preterm mice crossed a similar distance while in the open field, confirming similar levels of locomotor activity (Welch’s t-test Term/MFP Term vs Preterm p=0.86, t=0.18, df=52.07; N=30 Term, 25 MFP Term and 35 Preterm mice). **K)** Term and preterm mice had comparable preference for the walls of the open field arena, indicating comparable exploratory drive (t-test p=0.44, t=0.78, df=88).

To prepare the mice for task training, adult term and preterm mice were implanted with headposts, and then gradually water restricted and habituated to the experimental setup and handlers over 7–10 days ^40^, after which the training began. To account for the effects of prenatal mifepristone exposure on behavior, we included an additional group of mice that were born term to dams injected with mifepristone on GD 18 (MFP Term, Fig. 2A). Mice were trained on the task in 2 daily sessions of 100 trials, where each session consisted of 50 randomized presentations of 120º and 240º oriented gratings each. 120º was paired with the delivery of 10 μl of water, while 240º had no consequence (Fig. 2B and C). Licks of the spout were recorded and quantified as Hits (licks to 120º), Misses (failures to lick to 120º), False Alarms (licks to 240º) and Correct Rejections (failures to lick to 240º), which were then used to quantify the discriminability [d’=z(Hits)-z(False Alarms)]. Mice were considered trained after reaching a d’ of 2 or more for 3 sessions in a row. In our task, Hits remained high throughout the training, while False Alarms gradually decreased (Fig. 2D and Supplementary Fig. 1), driving an increase in d’. In term mice from both groups, d’ increased in a linear fashion (linear regression R^2^ Term = 0.73, MFP Term = 0.93; Term vs MFP Term p = 0.32, F = 0.97, DFn = 1, DFd = 354) and most mice crossed the threshold within 2 weeks of training (Fig. 2E and 2F). While preterm mice also displayed an increase in d’, the slope of the learning curve was significantly different from that of term born mice (linear regression R^2^ Preterm = 0.48; Term/MFP term vs Preterm p < 0.0001, F = 25.16, DFn = 1, DFd = 32), and a fraction of them (17.24%) failed to reach the criterion within the 4000 trial limit (20 days of training; Fig. 2E and 2F; p < 0.0001, Χ^2^ test), confirming that preterm birth negatively impacts cognition. Naïve preterm mice had significantly higher errors of omission and lower Hit rates at the onset of training, which recovered as the training progressed (Fig. 2G and Supplementary Fig. 1; 2-way ANOVA birth x training p = 0.004, F_(1, 68)_ = 9.16; birth p = 0.003, F = 9.25; training p < 0.0001, F = 36.88; Sidak post hoc: Term/MFP Term vs Preterm naïve p < 0.0001, trained p = 0.9; N = 22 Term/19 MFP Term and 29 Preterm mice). However, their False Alarm rates (errors of commission) remained high throughout the training (Supplementary Fig. 1) and were significantly higher at the end of the training compared to both groups of term mice (Fig. 2H; 2-way ANOVA birth x training p = 0.002, F_(1, 68)_ = 10.30; birth p = 0.44, F = 0.6; training p < 0.0001, F = 203.8; Sidak post hoc: Term/MFP Term vs Preterm naïve p = 0.19, trained p = 0.01), indicating reduced behavioral response inhibition ^48^.

As our task is performed in freely moving mice (on a treadmill), we tested the locomotor activity of term and preterm mice in an open field using an automated approach ^49^ to determine if preterm mice have any impairments in locomotion that might have precluded them from performing well in the task (Fig. 2I).

We found no significant differences between both groups of term mice and preterm mice in locomotor activity (Fig. 2J; Welch’s t-test Term/MFP Term vs Preterm p = 0.86, t = 0.18, df = 52.07; N = 30 Term, 25 MFP Term and 35 Preterm mice), and preterm mice showed typical levels of preference for the walls of the open field arena (thigmotaxis, Fig. 2K; t-test p = 0.44, t = 0.78, df = 88), indicating intact exploratory activity ^50^.

Our results hence demonstrate that preterm mice have impaired performance in a visual discrimination task, despite intact baseline visual function, locomotor activity and exploratory behavior in the open field arena. As discrimination impairments in mouse models of neurodevelopmental conditions are often associated with impaired visual processing during task performance ^51,52^, we next characterized the processing of task cues in the V1 of trained term and preterm mice while the mice were engaged in the task.

### Processing and representation of task cues are impaired in the V1 of preterm mice

**Figure 3:**
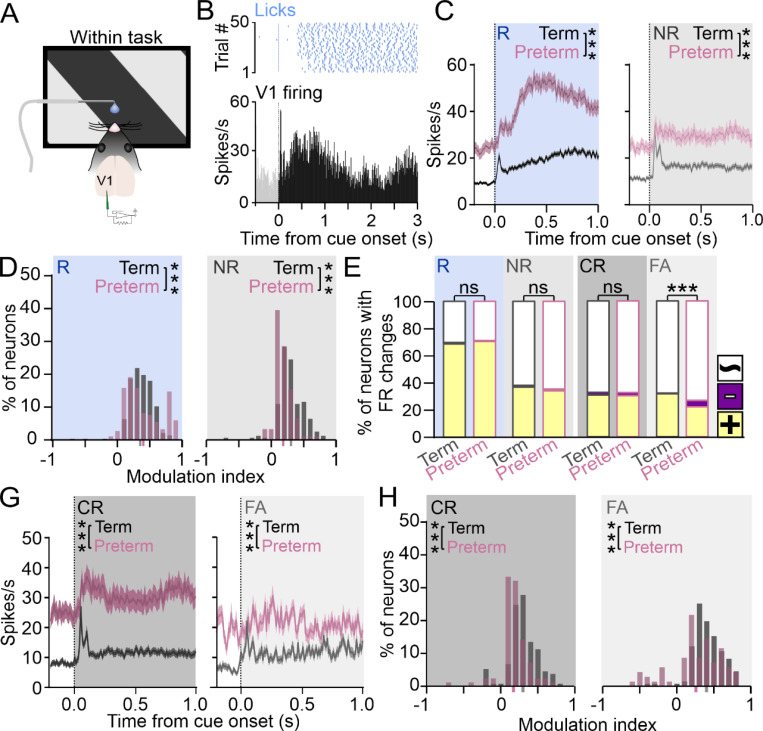
Processing and representation of task cues are impaired in the V1 of preterm mice. **A)** V1 activity of trained term and preterm mice was recorded while the mice were engaged in the task. **B)** Top: lick raster, and Bottom: peristimulus time histogram (PSTH) of a representative V1 neuron showing the transient and sustained components of the response during the cue presentation. **C)** PSTHs of V1 neurons signifcantly modulated by task cues revealed profound hyper-responsiveness of V1 neurons to the rewarded cue (left) and elevated firing rates during the presentation of the non-rewarded cue (right) in preterm mice (area under the curve (AUC): Term_R_=22±1.4, N=296 units from 12 mice and Preterm_R_=50.23±3.32, N=187 units from 10 mice, Welch’s t-test p<0.0001, t=7.81, df=251.8; Term_NR_=18.82±1.24, N=189 units and Preterm_NR_=34.52±2.55, N=121 units, p<0.0001, t=5.5, df=177). Data are represented as mean±SEM. **D)** Histogram of modulation indices of neurons in (C) revealed a significant shift to the right during in response to the rewarded cue (left) and a shift to the left in response to the non-rewarded cue (right), indicating stronger modulation by the rewarded cue and weaker by the non-rewarded cue. p<0.0001, Χ^2^ test. Mean modulation indices are indicated in grey (term) and pink (preterm) on X axis. **E)** Fractions of neurons significantly modulated by task cues are not significantly different between term and preterm mice, but the representation of the non-rewarded cue is significantly reduced in preterm mice during error trials (FA). Colors indicate positive (yellow), negative (violet) and while (unmodulated) neurons. p<0.0001, Χ^2^ test. **G)** PSTHs of neurons significantly modulated by the non-rewarded cue during associated behavioral outcomes revealed elevated firing rates during Correct Rejections and an almost complete absence of evoked activity during the False Alarms (AUC: Term_CR_=14.08±1.78, N=149 units from 12 mice and Preterm_CR_=36±2, N=119 units from 10 mice, Welch’s t-test p<0.0001, t=9.44, df=195.2; Term_FA_=13.05±1.6, N=162 units and Preterm_FA_=25.08±2.14, N=110 units, Welch’s t-test p<0.0001, t=4.51, df=218.8). Data are represented as mean±SEM. **H)** Modulation of neurons by the non-rewarded cue was shifted to smaller values for both behavioral outcomes. p<0.0001, Χ^2^ test. Mean modulation indices are indicated in grey (term) and pink (preterm) on X axis.

Visual discrimination training increases the representation of and responsiveness to trained cues in the V1, both thought to support task acquisition ^42,43,53–55^. As preterm mice display impairments in task acquisition (Fig. 2), we asked if the processing and representation of task cues in the V1 of preterm mice are altered compared to term mice. To address this, we used *in vivo* electrophysiology to isolate neuronal responses to task cues in trained mice while they were engaged in the task (Fig. 3A). Firing of neurons in the V1 of trained term and preterm mice displayed the typical transient (early) and sustained (late) components, where the peak of the sustained component typically preceded the onset of licks, suggesting a premotor nature (Fig. 3B)^56^. When we compared the peristimulus time histograms (PSTHs) of neurons whose firing was significantly modulated by task cues, we found prominent transient responses to task cues in both groups of mice, with a significant elevation of both pre- and post-cue firing in preterm mice [Figure 3C and 3D; area under the curve (AUC): Term_R_=22 ± 1.4, N = 296 units from 12 mice and Preterm_R_=50.23 ± 3.32, N = 187 units from 10 mice, Welch’s t-test p < 0.0001, t = 7.81, df = 251.8; Term_NR_=18.82 ± 1.24, N = 189 units and Preterm_NR_=34.52 ± 2.55, N = 121 units, p < 0.0001, t = 5.5, df = 177). The sustained responses to the rewarded cue were higher overall in both groups of mice, with a further elevation in preterm mice (Fig. 3C, left). Elevated firing to the rewarded cue in preterm mice was context-specific, as PTSHs revealed similar levels of activity in both groups of mice outside of task context (cues presented in the absence of the reward delivery spout, Supplementary Fig. 2; Term_R_=15.35 ± 1.4, N = 53 units from 12 mice and Preterm_R_=17.56 ± 1.37, N = 111 units from 10 mice; Welch’s t-test p = 0.26, F_(110, 52)_=1.96). Further, elevated firing rates in preterm mice were unlikely due to licking during the task, as the number of licks per trial was only slightly elevated in preterm mice (Supplementary Fig. 3; # of licks per bout Term_R_=23.65 ± 2.23, Preterm_R_=28.48 ± 1.49, Term_NR_=8.37 ± 1.28, Preterm_NR_=13.31 ± 3.056; birth x cue p = 0.97, F_(1, 22)_ = 0.00065; birth p = 0.024, F = 5.85; cue p < 0.0001, F = 46.26). Mice in both groups licked significantly less and in shorter bouts during the non-rewarded cue (lick bout duration Term_R_=5.39 ± 0.48, Preterm_R_=6.07 ± 0.34, Term_NR_=4.6 ± 0.49, Preterm_NR_=4.73 ± 0.62; birth x cue p = 0.47, F_(1, 22)_ = 0.52; birth p = 0.49, F = 0.49; cue p < 0.01, F = 7.69). In agreement with increased V1 firing to the rewarded cue, a higher fraction of neurons in preterm mice was strongly modulated by the rewarded cue (Fig. 3D, left; p < 0.0001, Χ^2^ test). In contrast, the modulation by the non-rewarded cue was significantly weaker in preterm mice (Fig. 3D, right; p < 0.0001, Χ^2^ test). The fraction of neurons responsive to the cues was not significantly different between term and preterm mice (Fig. 3E, p = 0.92 and 0.84, Χ^2^ test), indicating intact representation of task cues in the V1 of trained preterm mice despite impaired processing.

As each cue in our task can result in two behavioral outcomes (correct/CR and error/FA; Fig. 2C), we next asked if weakened modulation of the non-rewarded cue in the V1 is specific to incorrect behavioral responses given their higher rate in trained preterm mice (Fig. 2H). We were unable to test if strengthened modulation of neurons to the rewarded cue in preterm mice was specific to Hits or Misses as trained mice typically had a 100% hit rate during the last session (Supplementary Fig. 1). While the number of neurons responsive to the non-rewarded cue during the Correct Rejections was equal in term and preterm mice, preterm mice had a deficit in the representation of the non-rewarded cue during the False Alarm trials (Fig. 3E, p = 0.96 and < 0.0001, Χ^2^ test), indicating that the non-rewarded cue failed to sufficiently activate the V1 neurons of preterm mice if the result of the trial was an incorrect response. PSTHs revealed elevated baseline firing in preterm mice during both behavioral outcomes of the non-rewarded cue presentations, with the cue-evoked firing almost completely absent during False Alarms in preterm mice (Fig. 3G; AUC: Term_CR_=14.08 ± 1.78, N = 149 units from 12 mice and Preterm_CR_=36 ± 2, N = 119 units from 10 mice, Welch’s t-test p < 0.0001, t = 9.44, df = 195.2; Term_FA_=13.05 ± 1.6, N = 162 units and Preterm_FA_=25.08 ± 2.14, N = 110 units, Welch’s t-test p < 0.0001, t = 4.51, df = 218.8). Modulation of firing in preterm mice was weaker overall during both behavioral outcomes (Fig. 3H, p < 0.0001 for both, Χ^2^ test), with a significant fraction of neurons negatively modulated during False Alarms.

Altogether, our data demonstrates impairments in the processing of task cues in preterm mice, with strengthened neuronal responsivity to the rewarded cue and weakened responsivity to the non-rewarded cue. Preterm mice further have a deficit in the representation of the non-rewarded cue in the V1 during trials that result in an incorrect behavioral response. In combination with increased licking during the task performance (Supplementary Fig. 3) and reduced behavioral inhibition (Fig. 2H), processing of task cues in the V1 of preterm mice is reminiscent of cue processing in the adolescent brain ^29,30^, which is strongly reward-driven. Our results hence suggest that preterm birth impedes the maturation and/or function of brain areas that support the behavioral transition between adolescence and adulthood, such as the prefrontal cortex (PFC)^12,57,58^. Furthermore, learning-driven modulation of V1 processing is at least in part mediated by the PFC ^43,59^, so we next examined the neuronal responses to task cues in term and preterm PFC.

### ACC and ACC→V1 circuit in preterm mice are hyper-responsive to the rewarded cue

**Figure 4:**
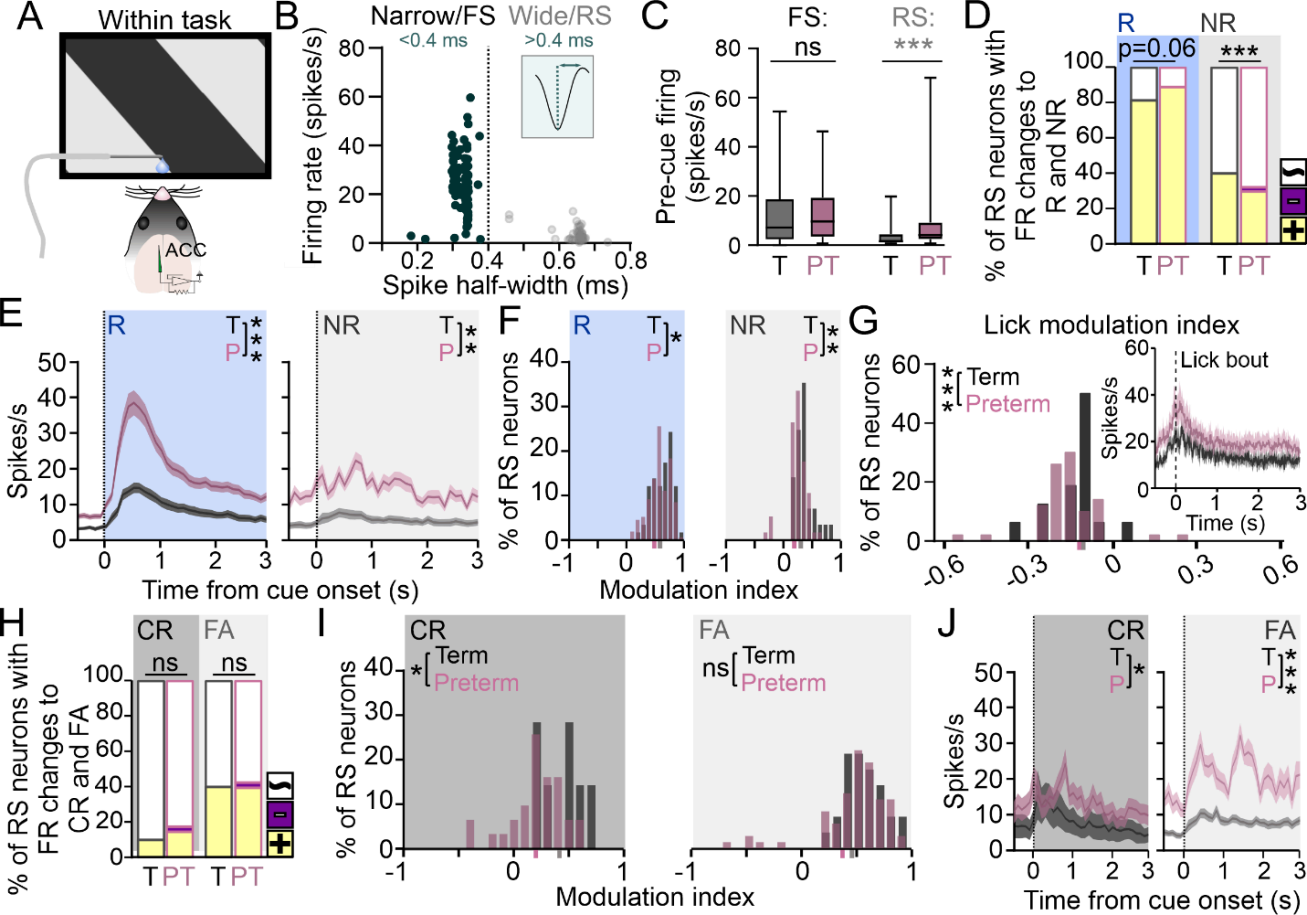
ACC of preterm mice is hyperactive and strongly reward-driven. **A)** ACC activity of trained term and preterm mice was recorded while the mice were engaged in the task. **B)** Isolated neurons were sorted into putative fast-spiking (FS, narrow waveform) and regular-spiking (RS, wide waveform) based on waveform width, with most narrow waveform units (<0.4 ms) had high firing rates. **C)** Mean firing rates of isolated FS and RS spiking units revealed increased firing rates of RS neurons in the ACC of preterm mice (FS Term=12.18±0.98, Preterm=12.15±0.96 spikes/s; N=131 and 134 units from 12 term and 10 preterm mice; RS Term=2.8±0.5, Preterm=7.4±0.8 spikes/s, N=63 and 154 units; Mann-Whitney t-test p<0.0001, U=3797). **D)** Representation of the rewarded cue is marginally increased, and that of the non-rewarded cue is significantly decreased in RS ACC neurons of preterm mice (p=0.06 and <0.0001, X^2^ test). Colors indicate positive (yellow), negative (violet) and while (unmodulated) neurons. **E)** PSTHs revealed robust activation of neurons in response to the rewarded cue (left) and highly irregular responses (right) to the non-rewarded cue in preterm mice (AUC: Term_R_ =26±3.61, N=57 units from 12 mice; Preterm_R_=61.29±10.10, N=151 units from 10 mice; Welch’s t-test p=0.0013, t=3.21, df=182.8; Term_NR_=18±2.36, N=28 units from 12 mice and Preterm_NR_=48.81±6.11, N=56 units from 12 mice; Welch’s t-test, p<0.0001, t=4.57, df=69.54). Data are represented as mean±SEM. **F)** Modulation by both cues is weaker in preterm mice (p=0.02 and 0.003, Χ^2^ test), with values of their modulation indices shifted to the left. Mean modulation indices are indicated in grey (term) and pink (preterm) on X axis. **G)** Modulation of RS unit firing by licks revealed that most isolated neurons ramp-up their firing rates prior to lick onset, resulting in negative modulation indices, which were significantly shifted to the left in preterm mice (p=0.0001, Χ^2^ test). Inset: PSTH of units modulated by licks, with the onset of lick bout at 0 s. Data are represented as mean±SEM. **H)** Representation of the non-rewarded cue during both behavioral outcomes is similar in term and preterm mice, with higher activation of neurons during the False Alarm trials (p=0.25 and 0.95, X^2^ test). Colors indicate positive (yellow), negative (violet) and while (unmodulated) neurons. **I)** Modulation of RS neurons by the non-rewarded cue is weaker during the Correct Rejection trials in preterm mice, with modulation index values significantly shifted to the left (p=0.025 and 0.44, X^2^ test). Mean modulation indices are indicated in grey (term) and pink (preterm) on X axis. **J)** PSTHs revealed highly irregular firing to the non-rewarded cue during both behavioral outcomes in preterm mice, with significantly elevated firing rates AUC: Term_CR_=21.73±3.39, N=7 units from 12 mice and Preterm_CR_=37.28±5.94, N=31 units from 10 mice; p=0.03, t=2.28, df=34.40; Term_FA_=21.58±2.12, N=28 units from 12 mice; Preterm_FA_=57.15±8.9, N=72 units from 10 mice; p=0.0002, t=3.85, df=78.46). Data are represented as mean±SEM.

Within the PFC, anterior cingulate cortex (ACC) sends and receives dense glutamatergic projections to and from the V1 ^60,61^. Neurons in the ACC are not responsive to sensory cues in naïve mice, but develop robust context-specific responsivity to sensory cues during training ^62–64^. Further, optogenetic activation of ACC neurons that project to the V1 (ACC→V1) evokes an increase in V1 firing rates and promotes behavioral performance in visual tasks ^59,60^, while inactivating ACC impairs visual discrimination (Supplementary Fig. 4)^48^, suggesting that ACC exerts top-down modulation of cue processing in the V1 of trained mice. We therefore hypothesized that cue processing impairments in the V1 of preterm mice might reflect an impairment of their ACC function during task performance. To test this, we recorded the activity of the ACC while the mice were engaged in the task (Fig. 4A) and sorted the isolated neurons by their waveform width into narrow, putative fast-spiking (FS) interneurons and wide, regular spiking (RS) putative pyramidal neurons, respectively (Fig. 4B)^65^. As expected, putative FS interneurons had higher firing rates overall in both groups of mice (Term = 12.18 ± 0.98, Preterm = 12.15 ± 0.96 spikes/s; N = 131 and 134 units from 12 term and 10 preterm mice). However, the firing rate of RS neurons in preterm mice was significantly elevated (Fig. 4C; Term = 2.8 ± 0.5, Preterm = 7.4 ± 0.8 spikes/s, N = 63 and 154 units; Mann-Whitney t-test p < 0.0001, U = 3797), so we first analyzed their responses.

The rewarded cue was represented marginally more in preterm than in term ACC RS neurons, while the non-rewarded cue representation was significantly weaker (Fig. 4D, p = 0.06 and < 0.0001, X^2^ test), with neurons robustly activated during the rewarded cue presentation in preterm mice, evident in their elevated cue-evoked firing rates (Fig. 4E, left; AUC: Term_R_=26 ± 3.61, N = 57 units from 12 mice; Preterm_R_=61.29 ± 10.10, N = 151 units from 10 mice; Welch’s t-test p = 0.0013, t = 3.21, df = 182.8). While responses during the non-rewarded cue presentation were also elevated in preterm mice (Fig. 4E, right: AUC: Term_NR_=18 ± 2.36, N = 28 units from 12 mice and Preterm_NR_=48.81 ± 6.11, N = 56 units from 12 mice; Welch’s t-test, p < 0.0001, t = 4.57, df = 69.54), the PSTHs revealed an irregular firing pattern with barely distinguishable cue-evoked peak in firing (Fig. 4E, right). However, RS neurons were modulated less by both cues in preterm mice (Fig. 4F, p = 0.02 and 0.003, Χ^2^ test), likely due to increased baseline firing. The increased firing of RS neurons in preterm mice was not due to increased licking (Supplementary Fig. 3) as licks negatively modulated neuronal firing in both groups of mice, and more so in preterm mice (Fig. 4G; p = 0.0001, Χ^2^ test). PSTHs to licks (Fig. 4G, inset) further revealed that the ramp-up of RS neuron activity occurs prior to the onset of the lick bout, indicating that most of the isolated neurons are responding to sensory input ^48^. Altogether, our data demonstrated elevated firing, increased reward cue-related activity and weakened representation of the non-rewarded cue in the ACC of preterm mice, similar to what we found in their V1 (Fig. 3) and indicating that neuronal activity during task performance in preterm ACC is strongly reward-driven.

Weakened representation of the non-rewarded cue in the V1 of preterm mice is specific to False Alarm trials, so we next tested if that is the case in the ACC as well. We compared the fraction of ACC RS neurons significantly modulated by the non-rewarded cue during Correct Rejections and False Alarms in term and preterm mice and, to our surprise, found no significant differences between term and preterm mice (Fig. 4H; p = 0.25 and 0.95, X^2^ test). RS neurons in both groups of mice were strongly activated by the non-rewarded cue during error trials, suggesting that their elevated activity instructs behavioral response (lick). However, the modulation of ACC RS neurons in preterm mice was shifted towards lower values during Correct Rejections (Fig. 4I; p = 0.025, X^2^ test), indicating an outcome-specific impairment of the modulation of RS neurons in the ACC of preterm mice. Similar to cue-evoked activity (Fig. 4E), the activity of ACC RS neurons in preterm mice during both outcomes was significantly higher, albeit highly irregular (Fig. 4K; AUC: Term_CR_=21.73 ± 3.39, N = 7 units from 12 mice and Preterm_CR_=37.28 ± 5.94, N = 31 units from 10 mice; p = 0.03, t = 2.28, df = 34.40; Term_FA_=21.58 ± 2.12, N = 28 units; Preterm_FA_=57.15 ± 8.9, N = 72 units; p = 0.0002, t = 3.85, df = 78.46).

**Figure 5:**
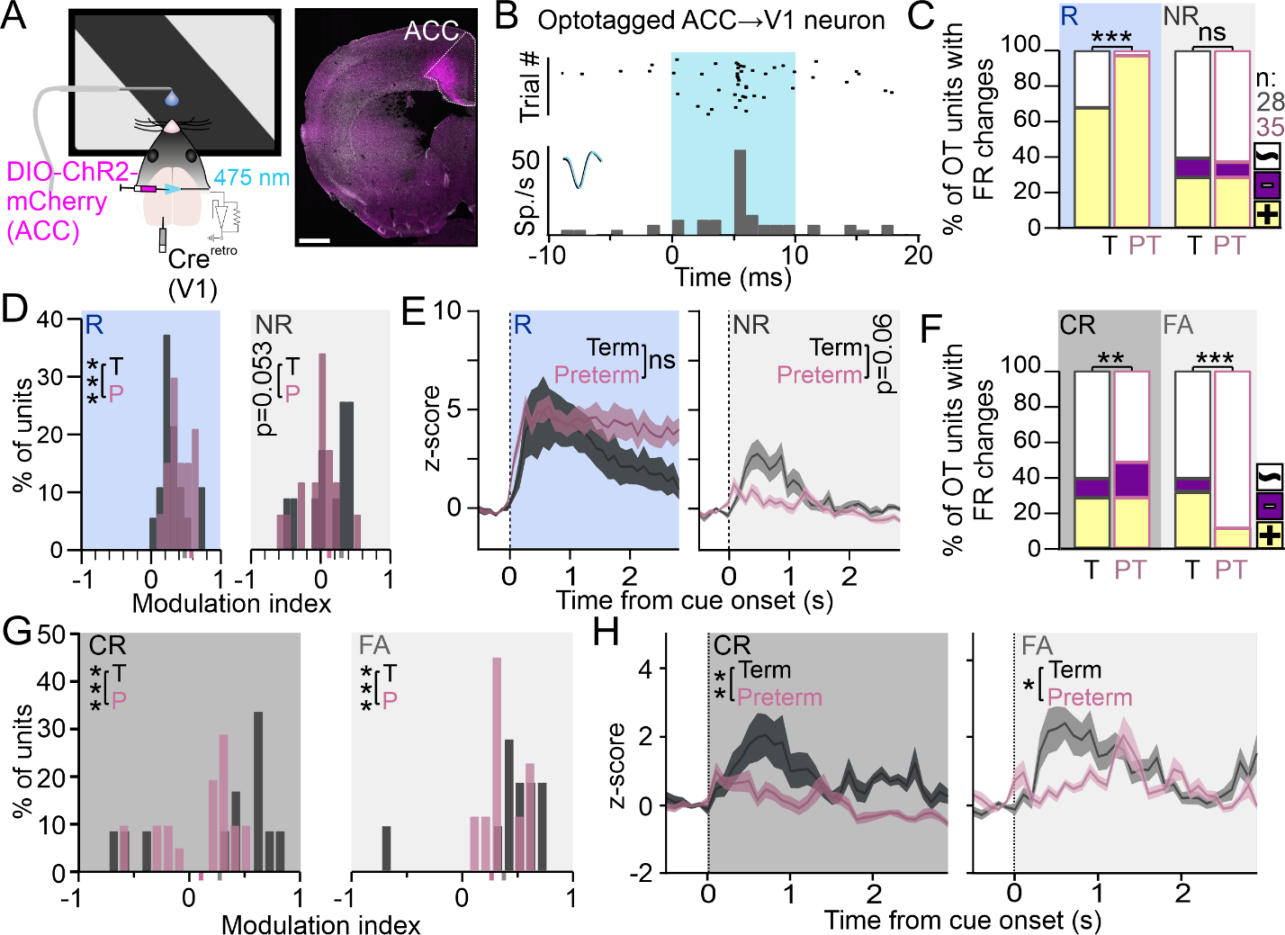
V1-projecting ACC neurons of preterm mice have a deficit in the representation of the non-rewarded cue during error trials. **A)** ACC→V1 neurons were labeled using AAV-Cre^retro^ and DIO-ChR2-mCherry and their activity recorded during task performance (left). Representative image of labeled neurons (magenta) with ACC indicated. Nuclei are labeled with DAPI (white). Scale bar: 500 μm. **B)** Representative raster and PSTH of an optotagged ACC→V1 neuron, with robust, regular and short latency firing in response to a 10 ms pulse of blue (473 nm) laser (indicated with blue rectangle). **C)** Rewarded cue activates almost all ACC→V1 neurons in preterm mice, whereas the non-rewarded cue activates an equal fraction of neurons in term and preterm mice (p<0.0001 and p=0.78, X^2^ test). Colors indicate positive (yellow), negative (violet) and while (unmodulated) neurons, and the numbers the total number of neurons isolated from both groups of mice. **D)** Modulation indices of ACC→V1 neurons by the rewarded cue in preterm mice are shifted to the right, indicating stronger modulation (p<0.0001, X^2^ test). Modulation indices by the non-rewarded cue were marginally lower in preterm mice (p=0.053, X^2^ test). Mean modulation indices are indicated in grey (term) and pink (preterm) on X axis. **E)** z-scored PSTHs revealed a faster ramp-up and slower ramp-down of ACC→V1 neurons after the presentation of the rewarded cue in preterm mice, with no significant difference in the firing rates between term and preterm mice (left; AUC: Term_R_=8.82±1.72, N=17 units from 7 mice, Preterm_R_=12.51±1.41, N=34 units from 6 mice, Welch’s t-test p=0.11, t=1.66, df=36.84). PSTHs of responses evoked by the non-rewarded cue (right) revealed an almost absent peak in activity in preterm mice, with marginal differences in the overall firing rate (AUC: Term_NR_=2.67±0.58, N=12 units, Preterm_NR_=1.28±0.39, N=18 units, Welch’s t-test p=0.06, t=1.99, df=20.35). Data are represented as mean±SEM. **F)** Representation of the non-rewarded cue during the associated behavioral outcomes is severely impaired in ACC→V1 neurons of preterm mice, with more suppressed neurons during correct responses and very few neurons activated by the non-rewarded cue during errors (p=0.008 and <0.0001, Χ^2^ test). Colors indicate positive (yellow), negative (violet) and while (unmodulated) neurons. **G)** Modulation indices of ACC→V1 neurons in preterm mice are shifted to the left, indicating weakened modulation by the non-rewarded cue during both behavioral outcomes (p<0.0001 for both, Χ^2^ test). Mean modulation indices are indicated in grey (term) and pink (preterm) on X axis. **H)** z-scored PSTHs to the non-rewarded cue revealed almost absent peaks in evoked activity in ACC→V1 neurons during both behavioral outcomes (AUC: Term_CR_=2.56±0.54, N=12 units and Preterm_CR_=0.65±0.3, N=20 units, Welch’s t-test p=0.006, t=3.12, df=17.78; Term_FA_=3.12±0.48, N=11 units and Preterm_FA_=1.86±0.29, N=9 units, Welch’s t-test p=0.039, t=2.25, df=15.88). Data are represented as mean±SEM.

Our bulk recordings, however, did not isolate the activity of ACC RS neurons that project to the V1 (ACC→V1), making it difficult to draw hypotheses about the circuit origins of impaired processing in the V1 of preterm mice. To do that, we used an intersectional viral approach to optogenetically label the ACC→V1 neurons, where we injected the retrograde AAV2 variant encoding the Cre-recombinase into the V1 ^66^ and DIO-ChR2-mCherry into the ACC, which resulted in dense labeling in the ACC (Fig. 5A). We used 10 ms pulses at 473 nm after the behavioral sessions ended to elicit the ChR2-mediated responses and isolate the ACC→V1 neurons (short latency responses with > 50% reliability, Fig. 5B) ^67^. Unlike the RS neurons isolated through bulk recordings of activity (Fig. 4), ACC→V1 neurons in term and preterm mice had comparable baseline firing rates (Term = 6.47 ± 1.04, N = 28 units from 7 mice; Preterm = 8.56 ± 2.001 spikes/s, N = 35 units from 6 mice; p = 0.36, Welch’s t-test, t = 0.93, df = 50.11). Interestingly, we found that almost all isolated ACC→V1 neurons in preterm mice were responsive to the rewarded cue, unlike in term mice (Fig. 4C; p < 0.0001, X^2^ test), and had stronger positive modulation by the rewarded cue despite no change in the firing rates (Fig. 5D and 5E; 5D: p < 0.0001, X^2^ test; 5E: AUC Term_R_=8.82 ± 1.72, N = 17 units from 7 mice, Preterm_R_=12.51 ± 1.41, N = 34 units from 6 mice, Welch’s t-test p = 0.11, t = 1.66, df = 36.84). ACC→V1 neurons were equally responsive to the non-rewarded cue in both groups of mice (Fig. 5C, p = 0.78, X^2^ test), but had marginally weaker modulation (Fig. 5D, p = 0.053, X^2^ test) and firing rates (Fig. 5E; AUC Term_NR_=2.67 ± 0.58, N = 12 units, Preterm_NR_=1.28 ± 0.39, N = 18 units, Welch’s t-test p = 0.06, t = 1.99, df = 20.35), with an almost absent cue-evoked peak (Fig. 5E). Hence, ACC→V1 neurons in preterm mice are strongly reward-driven, similar to other neurons in the ACC.

As preterm mice have a stronger impairment in non-rewarded cue processing in the V1 during trials that result in an incorrect response, we quantified the responses of ACC→V1 neurons to the non-rewarded cue during different behavioral outcomes. Unlike other RS neurons in the ACC, ACC→V1 neurons in term mice were equally activated by the non-rewarded cue during both behavioral outcomes, suggesting their sensory nature (Fig. 5F). However, preterm mice had an increased fraction of suppressed neurons during Correct Rejections and a severe deficit in the fraction of ACC→V1 neurons that are positively modulated by the non-rewarded cue during the False Alarms (Fig. 5F, p = 0.008 and < 0.0001, Χ^2^ test). The deficit was not as severe as in the V1 (Fig. 3), suggesting other sources of V1 modulation during False Alarm trials. Further, ACC→V1 neurons had weaker modulation during both behavioral outcomes (Fig. 5G; p < 0.0001 for both, Χ^2^ test) and significantly impaired firing, with an almost absent cue-evoked peak in activity during both outcomes in preterm mice (Fig. 5H; AUC: Term_CR_=2.56 ± 0.54, N = 12 units and Preterm_CR_=0.65 ± 0.3, N = 20 units, Welch’s t-test p = 0.006, t = 3.12, df = 17.78; Term_FA_=3.12 ± 0.48, N = 11 units and Preterm_FA_=1.86 ± 0.29, N = 9 units, Welch’s t-test p = 0.039, t = 2.25, df = 15.88).

Altogether, our results demonstrate a similar deficit in the representation and processing of the non-rewarded cue in the V1 and in the ACC→V1 neurons that connect the two areas and exert top-down modulation. We further found hyper-responsiveness to the rewarded cue in all recorded neurons, either in form of firing rates (RS neurons) or representation (ACC→V1 neurons), suggesting circuit disinhibition during reward cue processing in preterm mice. As prefrontal fast-spiking (FS) interneurons powerfully inhibit circuit activity ^68^, we compared their responses to task cues in the ACC of term and preterm mice.

#### Prefrontal FS interneurons in preterm mice have blunted responses to the rewarded cue and elevated activity during error trials

**Figure 6:**
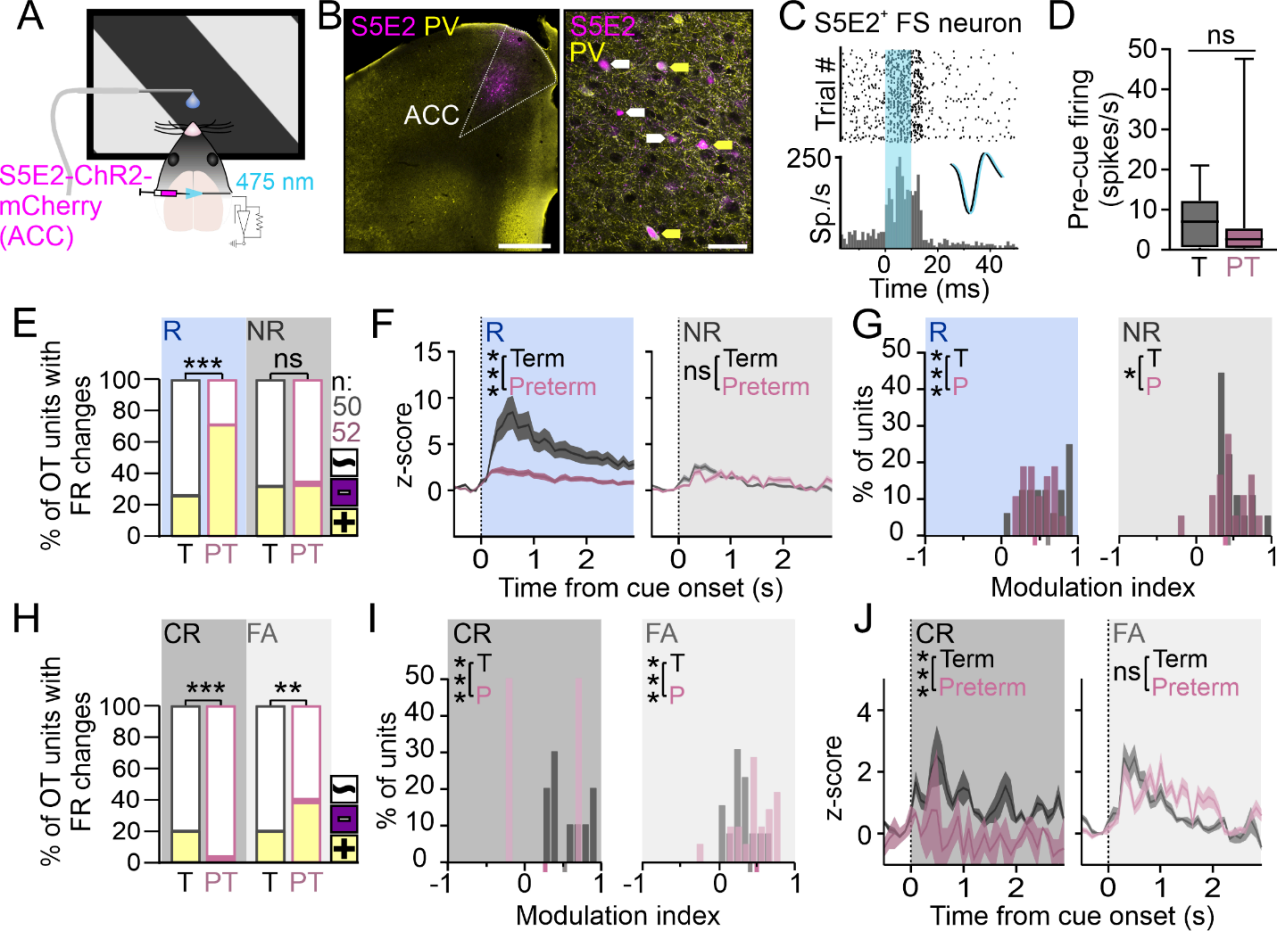
Optogenetically identified prefrontal FS neurons show impaired representation of task cues in preterm mice. **A)** FS neurons were labeled using S5E2-ChR2-mCherry and their activity recorded during task performance. **B)** Representative image of labeled neurons (magenta) with ACC indicated. Sections were counterstained with anti-Parvalbumin (PV) antibody (yellow). Yellow arrows indicate neurons with overlapping signals, white arrows indicate neurons with only mCherry fluorescence. Scale bars: 400 and 100 μm. **C)** Representative raster and PSTH of an optotagged S5E2^+^ FS neuron, with robust, rhythmic firing in response to a 10 ms pulse of blue (473 nm) laser (indicated with blue rectangle). **D)** Pre-cue firing of isolated neurons was not significantly different between term and preterm mice (Term=4.03±0.83 spikes/s, Preterm=4.48±1.2 spikes/s; Welch’s t-test p=0.76, t=0.3, df=89.14; N=50 units from 5 term and 52 units from 4 preterm mice). **E)** Rewarded cue activates a larger fraction of FS interneurons in preterm mice, with no differences in the representation of the non-rewarded cue (p<0.0001 and p=0.73, Χ^2^ test). Colors indicate positive (yellow), negative (violet) and while (unmodulated) neurons. **F)** z-scored PSTHs revealed a significant deficit in the activity of preterm FS interneurons evoked by the rewarded cue (AUC: Term_R_=13.36±1.36, N=13 units, Preterm_R_=4.03±0.68, N=37 units; Welch’s t-test p<0.0001). Data are represented as mean±SEM. **G)** Modulation indices of FS neurons by both cues were shifted to the left (p<0.0001 and p=0.034, Χ^2^ test). Mean modulation indices are indicated in grey (term) and pink (preterm) on X axis. **H)** Representation of the non-rewarded cue is severely impaired in preterm mice during both behavioral outcomes, with very few neurons activated during correct responses and more neurons activated during errors compared to term mice (<0.0001 and p=0.0006, Χ^2^ test). Colors indicate positive (yellow), negative (violet) and while (unmodulated) neurons. **I)** Modulation indices of FS unit during behavioral outcomes associated with the non-rewarded cues revealed significantly impaired modulation by the non-rewarded cue during correct responses (Correct Rejections) and stronger modulation during errors (False Alarms) in preterm mice (p<0.0001 for both, Χ^2^ test). Mean modulation indices are indicated in grey (term) and pink (preterm) on X axis. **J)** PSTHs revealed almost absent cue evoked responses during Correct Rejections (AUC: Term_CR_=3.1±0.47, N=10 units, Preterm_CR_=0.33±0.24, N=2 units; Welch’s t-test p<0.0001). Responses to the non-rewarded cue during False Alarms were irregular and non-selective, but not significantly different between term and preterm mice (AUC: Term_FA_=2.14±0.3, N=10 units, Preterm_FA_=2.8±0.41, N=21 units; Welch’s t-test p=0.2). Data are represented as mean±SEM.

Cortical fast-spiking (FS), Parvalbumin (PV) interneurons provide strong perisomatic inhibition to pyramidal neurons and their functional maturation in adolescence is thought to facilitate the behavioral transition to adulthood ^69–72^. In support of this notion, chemogenetic activation of prefrontal PV interneurons suppresses impulsive action, a hallmark of adolescent behavior ^73,74^. PV interneurons are frequently impaired in animal models of prematurity ^18,21,75–77^ which, in combination with our results demonstrating hyperexcitability of prefrontal pyramidal neurons (Fig. 4C), led us to hypothesize that prefrontal PV interneurons in preterm mice are hypoactive. To test this, we used a transcriptional enhancer S5E2 ^78^ to reliably transduce FS interneurons in the ACC with ChR2 for optotagging (Fig. 6A and 6B). We chose this approach as FS interneurons can remain undetected during spike sorting of bulk electrophysiological recordings due to their low spontaneous firing rates and weak labeling with PV-Cre mouse line ^79,80^. S5E2-mCherry only partially colocalized with Parvalbumin (PV) signal in the ACC, which was significantly weaker compared to other cortical areas (Fig. 6B), as previously reported. As expected, 10 ms pulses of blue light evoked strong rhythmic firing in transduced neurons (Fig. 6C)^67^, with all isolated neurons having a narrow waveform (average spike half-width: 0.33 ± 3.2×10^−6^ ms, N = 50 units from 5 term mice and 52 units from 4 preterm mice). The overall pre-cue firing rate of optotagged FS units was significantly lower than that of FS units isolated through spike sorting of bulk electrophysiological data (Fig. 4B and 4C; Term/Preterm narrow-spiking units = 12.16 ± 0.69 spikes/s vs Term/Preterm optotagged S5E2^+^ units = 4.26 ± 0.74 spikes/s; Welch’s t-test p < 0.0001, t = 7.82, df = 271.8), with no significant differences between term and preterm FS units (Fig. 6D; Term = 4.03 ± 0.83 spikes/s, Preterm = 4.48 ± 1.2 spikes/s; Welch’s t-test p = 0.76, t = 0.3, df = 89.14). To our surprise, we found that a larger fraction of optotagged units was positively modulated by the rewarded cue in preterm mice, with no differences in the responsivity to the non-rewarded cue (Fig. 6E; p < 0.0001, Χ^2^ test). However, when we plotted the PSTHs of isolated units, we found a stark deficit in the activity evoked by the rewarded cue in preterm mice (Fig. 6F, left; AUC: Term_R_=13.36 ± 1.36, N = 13 units, Preterm_R_=4.03 ± 0.68, N = 37 units; Welch’s t-test p < 0.0001) and weaker modulation values (Fig. 6G, left; p < 0.0001, Χ^2^ test) supporting that the hyper-responsiveness to the rewarded cue in preterm mice might be due to the *hypo*responsiveness of FS interneurons. There were no differences in firing evoked by the non-rewarded cue (Fig. 6F, right; AUC: Term_NR_=2.88 ± 0.33, N = 16 units, Preterm_NR_=2.89 ± 0.44, N = 18 units; Welch’s t-test p < 0.0001), but the firing in preterm mice appeared irregular, much like in other ACC neurons (Fig. 4F and 5D) and the modulation was slightly lower (Fig. 6G, right; p = 0.034, Χ^2^ test).

To test for differences in the activity of FS interneurons during specific behavioral outcomes, we compared their activity in term and preterm mice during Correct Rejections and False Alarms (Fig. 6H). While an equal fraction of FS interneurons was activated during both outcomes in term mice, there was a pronounced imbalance in preterm mice, with almost no units that were activated by the non-rewarded cue during the Correct Rejections and significantly more units activated during False Alarms (Fig. 6H; p < 0.0001 and p = 0.0006, Χ^2^ test). The modulation values reflected this distribution, with weaker modulation during the Correct Rejections and stronger during the False Alarms in preterm mice (Fig. 6I; p < 0.0001 for both, Χ^2^ test). Indeed, PSTHs revealed an almost complete absence of cue-evoked activity during Correct Rejections in preterm mice (Fig. 6J, left; AUC: Term_CR_=3.1 ± 0.47, N = 10 units, Preterm_CR_=0.33 ± 0.24, N = 2 units; Welch’s t-test p < 0.0001), as well as irregular and persistent firing during the False Alarms which was not significantly different to that in term mice (Fig. 6J, right; AUC: Term_FA_=2.14 ± 0.3, N = 10 units, Preterm_FA_=2.8 ± 0.41, N = 21 units; Welch’s t-test p = 0.2). Altogether, our results demonstrated a severe deficit in the activation of FS interneurons during the presentation of the rewarded cue despite its increased representation. The representation of the non-rewarded cue was imbalanced during the behavioral outcomes associated with that cue, with weak activation during correct responses and stronger activation during errors, supporting that suppressed activity of ACC→V1 neurons during False Alarms stems from heightened inhibition.

### Life-long environmental enrichment fails to improve the learning trajectory of preterm mice

**Figure 7:**
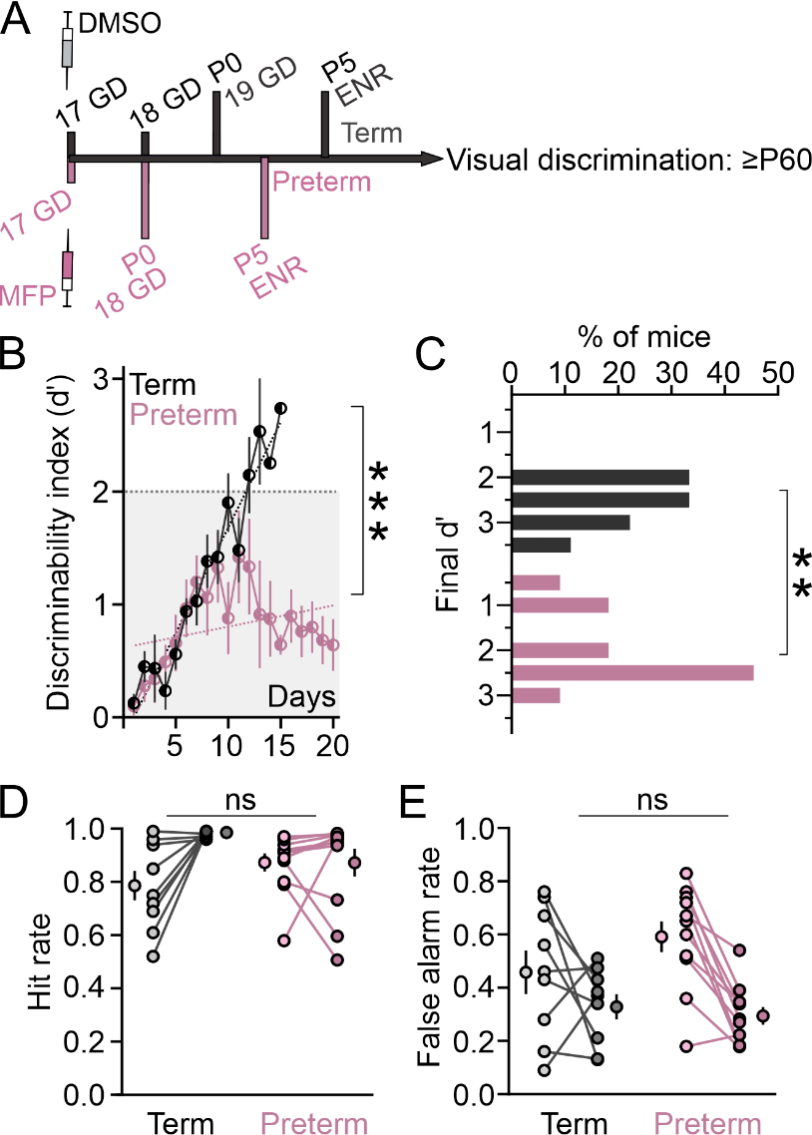
Lifelong environmental enrichment fails to improve the learning trajectory of preterm mice. **A)** Experimental timeline. Term and preterm mice were housed in enriched environment from postnatal day 5 (P5) until the end of the training. **B)** The learning trajectory of term mice reared in enriched environment was steep (R^2^ Term_ENR_=0.94, Preterm_ENR_=0.09; p<0.0001, F_(1, 31)_=63.11). Data are represented as mean±SEM of 9 term mice and 11 preterm mice. **C)** A significant proportion of preterm mice reared in enriched environment failed to reach the criterion, unlike term mice who reached very high values (p=0.004, Χ^2^ test). **D)** Hit rates were not significantly different between term and preterm mice reared in enriched environment (2-way ANOVA: training x birth interaction p=0.021, F_(1, 18)_=6.45; birth p=0.77, F=0.084, training p=0.02, F=6.339; no significant differences between term and preterm mice in the first or last session, Šídák’s multiple comparisons test). **E)** False Alarm rates were not significantly different between term and preterm mice reared in enriched environment (2-way ANOVA: training x birth interaction p=0.13, F_(1, 18)_=2.49; birth p=0.42, F=0.7, training p=0.0008, F=16.1; no significant differences between term and preterm mice in the first or last session, Šídák’s multiple comparisons test).

Adult preterm mice exhibit impaired learning trajectory and reduced behavioral response inhibition during visual discrimination learning, as well as heightened neuronal responsivity to the rewarded cue in the visual and prefrontal cortices, suggesting that their top-down circuits that modulate sensory associative learning are in an adolescent-like maturational state ^29,30,74^. We therefore tested if environmental enrichment (ENR), a classical paradigm that promotes sensory maturation and rescues cognitive deficits in animal models of prematurity-related brain injury, improves visual discrimination learning in preterm mice ^21,32,35,81,82^. To do this, we housed timed-pregnant dams with their term and preterm litters from postnatal day 5 (P5) to weaning (P28) in standard rat cages supplemented with a running wheel, multiple play and chew objects (such as gnawing sticks and tunnels), as well as a non-related dam with an age-matched litter for social enrichment. The object location was shuffled in the cage every 2–3 days. Prior to P5, pups were housed in a standard cage with enrichment for the dams in form of igloos, extra nesting material and sunflower seeds. After weaning, term and preterm mice were housed in groups of 2–3 in standard cages supplemented with an igloo, small running platform and gnawing sticks. When mice reached adulthood (> 2 months of age), they were prepared for the visual discrimination task, water-restricted and trained to criterion (Fig. 7A). Term mice responded positively to the enrichment, with a steep learning curve whose slope was significantly different to that of standardly housed (STD) term mice (Fig. 7B; compare with Fig. 2E; R^2^ Term_ENR_=0.94, Term_STD_=0.73; p = 0.02, F_(1,24)_ = 6.05) and a higher proportion of mice reaching high d’ values (Fig. 7C, compare with Fig. 2F; p < 0.0001, Χ^2^ test). However, ENR had no effect on the overall learning trajectory of preterm mice, who continued to display a flatter learning trajectory (Fig. 7B; R^2^ Preterm_ENR_=0.09; compared to Term_ENR_ p < 0.0001, F_(1,31)_ = 63.11) and frequent failure to reach the criterion (Fig. 7C; p = 0.004, Χ^2^ test). Overall slope of learning curve was not significantly different between standardly reared preterm mice and those reared in enriched environment (Fig. 7B, compare with Fig. 2E; p = 0.18, F_(1,36)_ = 1.91). To our surprise, ENR resulted in a significantly higher fraction of preterm mice that failed to reach the criterion (Fig. 7C: 27.27% of mice, compare with Fig. 2F: 17.24%; p = 0.007, Χ^2^ test). There were no significant differences in hit rates or in false alarm rates between term and preterm mice raised in ENR (Fig. 7D and 7E). However, both were lower in naïve ENR term mice when compared with standardly reared mice (Fig. 7D and 7E; Hit rates: Term_STD_=0.94 ± 0.03 from 22 mice, Term_ENR_=0.78 ± 0.05 from 9 mice, Mann-Whitney t-test p = 0.01, U = 42; False alarm rates: Term_STD_=0.78 ± 0.04, Term_ENR_=0.46 ± 0.08, t-test, p < 0.0008, F_(8,21)_=1.47), suggesting that ENR suppressed overall motor output in our task. In line with this notion, training did not improve the hit rates in ENR preterm mice as it did in STD preterm mice (Fig. 7D; Preterm_STD_=0.98 ± 0.004 from 29 mice, Preterm_ENR_=0.86 ± 0.05 from 11 mice, Mann-Whitney t-test p < 0.0001, U = 27) and ENR preterm mice had lower false alarm rates at the end of the training when compared to STD preterm mice (Preterm_STD_=0.45 ± 0.04 from 29 mice, Preterm_ENR_=0.3 ± 0.03 from 11 mice, t-test p = 0.02. F_(28,10)_ = 3.08).

## Discussion

Through extensive electrophysiological characterization of primary visual (V1) and prefrontal cortices in adult preterm mice trained on a visual associative task, our study identified impaired representation and processing of behaviorally salient sensory cues as a potential driver of cognitive impairments common in preterm born children and adolescents. Increased neuronal responsiveness to the rewarded cue and diminished responses to the non-rewarded cue in preterm mice were present across brain areas and neuronal subtypes examined, with prominent deficits during error trials in prefrontal V1-projection neurons and during correct responses in prefrontal fast-spiking (FS) interneurons. As most preterm mice eventually learn to associate the task cues with correct behavioral outcomes, our results suggest an atypical neural mechanism of learning in the mature preterm brain. Indeed, heightened responsivity to the rewarded cue that we detected in the prefrontal cortex (PFC) is reminiscent of cue processing in the adolescent brain ^29^, suggesting that preterm birth impedes the function of circuits that support the transition to mature processing of behaviorally salient cues. It is still unclear if the prefrontal dysfunction of preterm mice is due to arrested or disrupted development. However, environmental enrichment, a commonly used developmental intervention that rescues arrested sensory maturation ^82^, is ineffective in improving the behavioral performance of preterm mice, arguing for the latter. While it is possible that the type of enrichment used in our study was not optimal for promoting the maturation of top-down areas, the improvement in performance of term mice reared in enriched environment compared to those reared in the standard one suggests that this is not the case. Future longitudinal studies can now test how preterm birth disrupts the maturational trajectory of the PFC.

Our results further demonstrated a substantial hyperactivity of PFC and V1 neurons in preterm mice, indicating a deficit in the activity of cortical interneurons. Indeed, the response of prefrontal FS interneurons to the rewarded cue was particularly blunted in preterm mice, suggesting that elevated responsiveness of prefrontal pyramidal neurons to the same cue is a consequence of dysfunctional perisomatic inhibition. However, neurons in the PFC and V1 of preterm mice are hyperactive even before the cue presentation, when FS interneurons do not show reduced firing rates, indicating a hypofunction of at least one other interneuron subtype in the cortex of preterm mice. Indeed, abnormalities in multiple interneuron subtypes have been reported in postmortem studies of prematurely born fetuses and in animal models of prematurity ^17,19,20,83^. Future electrophysiological studies can now address which interneuronal subtype is hypoactive at baseline in preterm PFC.

In case of blunted activation of FS interneurons during the rewarded cue, the identity of inputs that fail to sufficiently activate the FS interneurons is unclear. These inputs are unlikely to come from local pyramidal neurons as their responses to the rewarded cue in preterm mice are robust. Another major source of excitation to prefrontal FS interneurons is the mediodorsal thalamus (MDT), whose role in learning is well established ^84–86^. Optogenetic activation of MDT results in a reduction of putative pyramidal neuron firing rates due to strong feedforward inhibition mediated by MDT inputs onto FS interneurons ^68,87^. Our results point to these inputs being dysfunctional in preterm mice, resulting in elevated activity of prefrontal pyramidal neurons during cue presentation. This notion is in line with impairments in thalamic structure observed in preterm infants and adolescents ^13,14^ and the hypothesized role of thalamic dysfunction as a driver of impaired cortical function after preterm birth ^88^. In sensory areas of mice, preterm birth accelerates the refinement of thalamocortical inputs through serotonin signaling ^89^, but the functional impact of these structural changes is unclear. Preterm born infants and mice show accelerated maturation of neural activity in the V1 ^24,90^, indicating that preterm birth advances the maturation of sensory thalamic nuclei and their cortical target areas. However, it is unclear if prematurity has the same effect on higher order thalamic nuclei, such as MDT, whose cortical target is the PFC. We plan to address this in our future studies.

We previously found that juvenile preterm mice show sparser neuronal firing rates and increased size of putative inhibitory synapses in the V1 compared to term-born mice ^24^. Our current results indicate that these changes in the activity of V1 in preterm mice are likely transient, given the comparable neuronal firing rates of V1 neurons outside of task context in term and preterm mice. Furthermore, the baseline orientation selectivity is normal in preterm mice and the firing of V1 neurons is elevated only during task performance, indicating that visual processing deficits common after preterm birth are due to top-down modulation and not local V1 computations ^91,92^. Our findings of impaired responses to the non-rewarded cue in V1-projecting anterior cingulate cortex (ACC) neurons further support this notion and provide mechanistic insight into previous reports of top-down sensory processing impairments in preterm infants ^93–95^. Responses of V1-projecting ACC neurons to task cues in term mice were distinct from other putative pyramidal neurons in the PFC: while the representation of the rewarded cue was higher than that of the non-rewarded cue, both cues evoked responses of comparable magnitude in activated neurons, which we have not observed in other regular spiking (RS) PFC neurons. Robust activation of ACC→V1 neurons during both outcomes of the non-rewarded cue in term mice is in agreement with previous finding that implicated this projection in error monitoring and visual attention ^59^. Indeed, the response of ACC→V1 neurons to the non-rewarded cue in preterm mice is blunted, with very few neurons activated during the incorrect responses, suggesting an impairment in error monitoring in preterm mice. Our results hence support that impaired function of this top-down circuit may drive visual attention deficits that are common in the preterm population ^91,96–98^.

While our study identified potential circuit drivers of impaired neurodevelopmental outcomes after preterm birth, it did not test if optogenetic or chemogenetic manipulations of circuit- or cell type-specific activity improved behavioral performance of preterm mice in our task. Chemogenetic activation of prefrontal FS interneuron promotes behavioral response inhibition ^73^, but it is unlikely that chemogenetic or pharmacological approaches would have the same effect in preterm mice given the cue- and outcome-specific deficits in their activity. Such specificity could be achieved via optogenetic manipulation, but the translational impact of such approach is difficult to estimate. As previously discussed, environmental enrichment failed to improve the behavioral outcomes of preterm mice, casting doubt on efficacy of similar approaches for promoting neurocognitive outcomes in the preterm population ^32,99,100^. Previous research highlighted windows of increased plasticity and vulnerability during prefrontal development beginning shortly after birth ^12,101,102^. It is possible that targeted activity and/or circuit manipulations during these windows could improve neurocognitive outcomes after preterm birth, but the mechanistic impact of prematurity on the developmental trajectory of the PFC is unknown. This question must be addressed in future studies to facilitate inquiries into preventative measures or treatments for prematurity-related impairments in brain function that could be implemented as early as during infancy.

In conclusion, our results identify impairments in the representation and processing of sensory cues in the visual and prefrontal cortices of preterm mice, and provide further evidence of interneuron dysfunction in the preterm brain ^17,18,103^. Our study highlights the profound and persistent impact of preterm birth on the function of top-down circuits, and points to potential circuit mechanisms of increased risk of impaired neurodevelopment in the preterm population.

## Supplementary Material

Supplement 1
