# Supplementary material for: Adolescent-like Processing of Behaviorally Salient Cues in Sensory and Prefrontal Cortices of Adult Preterm-Born Mice
Source: Res Sq. 2024 Dec 11:rs.3.rs-5529783. Preprint. [Version 1] doi: 10.21203/rs.3.rs-5529783/v1 (PMC11661414; doi:10.21203/rs.3.rs-5529783/v1)
Supplement: Supplement 1 [file NIHPPRS5529783V1-supplement-1.pdf]

Supplementary Files

This is a list of supplementary files associated with this preprint. Click to download.

- SupplementaryFigures.docx
